# Increased frequencies of human Th-17 CD4+ T-cells and decreased T-regulatory cells in patients with early and advanced metabolic dysfunction-associated steatotic liver disease

**DOI:** 10.3389/fimmu.2025.1597204

**Published:** 2025-08-22

**Authors:** Elżbieta Supruniuk, Kamil Grubczak, Anna Parfieniuk-Kowerda, Robert Flisiak, Marcin Moniuszko, Jerzy Jaroszewicz, Adrian Chabowski, Magdalena Świderska

**Affiliations:** ^1^ Department of Physiology, Medical University of Bialystok, Bialystok, Poland; ^2^ Department of Regenerative Medicine and Immune Regulation, Medical University of Bialystok, Bialystok, Poland; ^3^ Department of Infectious Diseases and Hepatology, Medical University of Bialystok, Bialystok, Poland; ^4^ Department of Allergology and Internal Medicine, Medical University of Bialystok, Bialystok, Poland; ^5^ Department of Infectious Diseases and Hepatology, Medical University of Silesia, Bytom, Poland; ^6^ Medical Diagnostic and Microbiological Laboratory of Ludwik Rydygier Hospital in Suwalki, Suwalki, Poland

**Keywords:** metabolic dysfunction-associated steatotic liver disease, Th17 CD4+ T-cells, T-regulatory cells, fibrosis, IL-10, IL-17A, IL-22

## Abstract

**Background:**

Dysregulation of immune responses may influence the progression of metabolic dysfunction-associated steatotic liver disease (MASLD) to metabolic dysfunction-associated steatohepatitis (MASH). Our recent data suggest the role of Th17-related cytokines in fibrosis advancement in MASLD. Herein, we aimed to analyze T-regulatory and Th17-producing T-lymphocytes by flow cytometry with respect to MASLD progression.

**Methods:**

Extensive immunophenotyping was performed in a subset of 30 patients with MASLD diagnosed by elastography and ultrasonography and 15 healthy controls (HCs). Ex-vivo surface markers (CD4, CD25, CD127) and intracellular cytokine expressions (IL-10, IL-17, Foxp3, RORgt) were analyzed by flow cytometry (BD FACS-Calibur). Plasma concentrations of selected interleukins such as IL-10, IL22, and IL-17A were measured by ELISA.

**Results:**

19/30 (63%) of MASLD patients were diagnosed with steatosis with inflammation (advanced MASLD) as compared to simple steatosis (early MASLD) using elastography. The percentage of IL-17-producing cells among CD4(+) T-lymphocytes was two-fold more frequent (1.70% *vs*. 0.73%), while of T-regulatory cells (CD4+CD25+Foxp3+, T-regs) lower (3.57% *vs*. 6.56%) in advanced MASLD compared to HCs. This resulted in an aberrated ratio of Th17 to Tregs in MASLD (p=0.004). The frequency of T-regulatory cells (CD4+CD25+Foxp3+, Tregs) declined also in the advanced MASLD patients (3.57%) compared to the early stage disease (5.16%). Importantly, IL-10 and IL-17A serum levels positively correlated with CD4+IL-17+/CD4+CD25+Foxp3+ ratio. Plasma IL-10/IL-17A ratio and IL-10/IL-22 ratio significantly differed between F0 fibrosis *vs*. moderate (F2).

**Conclusions:**

The imbalance between Th17 and T-regulatory immune responses is present not only at cytokine level but also at a cellular level in MASLD. Especially in advanced disease, a higher percentage of IL-17 producing T-cells is coupled with the lower number of T-regulatory cells.

## 1 Introduction

Metabolic dysfunction-associated steatotic liver disease (MASLD) is a highly dynamic disease that affects more than 38% of the world’s general population, although its diagnostic techniques are still not fully adequate. Pathogenesis is closely linked to obesity and metabolic syndrome (1). Among patients with MASLD, approximately 30% will develop metabolic dysfunction-associated steatohepatitis (MASH) (2, 3) as a result of inflammation associated with the recruitment and activation of inflammatory cells in the liver with pre-existing steatosis (4). It is important to note that frequent exacerbations of MASLD to MASH result in the progression of type 2 diabetes, diseases of the cardiovascular system, liver cirrhosis, and hepatocellular carcinoma (HCC) (5, 6). Historically, MASLD diagnosis required liver biopsy to detect steatosis (7). However, this invasive procedure is associated with a risk of complications and variability due to the uneven distribution of lesions as it only assesses approximately 1 in 50,000 of the entire liver parenchyma (8, 9). Novel non-invasive modalities, including elastography, show the limited capability of differentiating between mild and moderate fibrosis, which is of importance for the selection of treatment (9, 10). There is no screening test, which will allow the routine determination of blood to detect irregularities. In order to prevent further progression of MASLD fast implementation of the principles of healthy diet as well as the application of appropriate treatment therapies is indispensable. Therefore, there is a strong need for new noninvasive markers of liver injury, especially to detect not only fibrosis, but also the advancement of inflammatory processes, particularly at less advanced stages of the disease (11).

CD4+ T cells play an important role in the inflammation and progression of liver fibrosis and chronic liver diseases, including MASLD. Among subsets of CD4+ T cells stimulate differentiation of Treg cells that mediate immune tolerance, and Th17 cells, which induce severe inflammation (12, 13). Th17 cells may contribute to rapid steatosis of the liver and accelerate progression from simple steatosis to steatohepatitis (14, 15). In the process of liver damage, the imbalance between the pro-inflammatory and anti-inflammatory mechanisms of congenital and acquired immunity is significant (6, 13, 16). Therefore, the change in the balance of Treg/Th17 cells may be an effective strategy for monitoring disease progression in patients with MASLD (13, 15, 16).

The aim of the study was to analyze the activity of regulatory T cells and Th17 determined by the cytometric method in patients with MASLD, as well as to establish the effect of Th17 immune response activity and associated cytokines on the progression of MASLD (early *vs*. advanced disease). In addition, the number of circulating lymphocytes producing IL-17A and IL-10 in the peripheral blood of patients with MASLD was assessed depending on the biochemical and histological activity of the disease.

## 2 Materials and methods

### 2.1 Patient characteristics

The experimental protocols and sample studies were approved by the Ethics Committee of the Medical University of Bialystok, Bialystok, Poland (permission number: R-I-002/93/2014, date of approval: 27.03.2014) and followed the internationally endorsed standards as stated in the Declaration of Helsinki. All patients were adults and were informed of the aim of the study. The clinical characteristics of the studied population are presented in **Table 1**.

**Table 1:** Clinical characteristics of MASLD, early MASLD, advanced MASLD patients, and healthy controls (HCs).

Parameter	MASLD n=30	Early MASLD n=11	Advanced MASLD n=19	HCs n=15
Sex (M/F)	16 M/14 F	5 M/6 F	11 M/8 F	6 M/9 F
Age (years)	47 (38 – 58)	53 (38 – 58)	44 (38 – 60)	36 (28 – 52)
WHR	0.94 (0.91 – 0.99)	0.91 (0.88 – 0.96)	0.97 (0.93 – 1.06)	0.76 (0.73 – 0.90)
Weight (kg)	86 (81 – 109)	86 (80 – 102)	92 (82 – 110)	68 (55 – 77)
Height (cm)	173 (162 – 182)	163 (161 – 182)	175 (164 – 182)	170 (168 – 176)
BMI (kg/m^2^)	30.05 (27.77 – 33.67)*	30.0 (25.96 – 33.95)	31.0 (27.99 – 33.58)	23.2 (19.5 – 24.5)
ALT (U/L)	57.0 (38.0 – 86.0)	38.0 (32.0 – 39.0)	84.0 (58.0 – 127.0)*#	35 (26 – 37)
AST (U/L)	35.0 (28.0 – 53.8)*	28.0 (19.0 – 34.0)	47.0 (35.0 – 69.0)*#	23 (19 – 28)
GGT (U/L)	42.0 (26.0 – 119.5)	36.0 (25.0 – 207.0)	43.0 (34.8 – 91.2)	–
Cholesterol (mg/dL)	198.0 (179.5 – 208.5)	173.5 (160.0 – 187.5)	205.0 (195.0 – 211.0)	158 (146 – 184)
LDL-C (mg/dL)	112.0 (99.3 – 151.8)	101.5 (97.5 – 115.5)	144.5 (109.8 – 180.0)	–
HDL-C (mg/dL)	52.0 (42.5 – 56.0)	50.0 (33.3 – 66.0)	52.0 (47.5 – 52.0)	–
Leukocytes (10^9^/L)	6.74 (5.78 – 8.90)	6.68 (5.78 – 9.40)	7.04 (5.42 – 8.77)	7.11 (6.00 – 9.01)
Erythrocytes (10^12^/L)	4.73 (4.53 – 5.22)	4.53 (4.37 – 4.88)	4.84 (4.66 – 5.33)	4.7 (3.9 – 4.9)
Platelets (10^9/^L)	204.5 (173.3 – 265.8)	257.0 (199.0 – 302.0)	189.0 (165 – 238)*#	320 (289 – 361)
Fibrinogen (mg/dL)	211.5 (203.0 – 294.3)	241.5 (199.9 – 283.0)	211.5 (204.5 – 309.5)	–
Prothrombin time (s)	11.0 (10.30 – 11.70)	11.30 (10.45 – 13.55)	10.80 (10.20 – 11.70)	–
INR	0.96 (0.916 – 1.04)	0.96 (0.92 – 1.05)	0.96 (0.91 – 1.04)	–
Prothrombin index	104.5 (93.5 – 115.5)	104.0 (94.0 – 116.0)	105.0 (93.0 – 117.0)	–
Total bilirubin (mg/dL)	0.62 (0.44 – 1.11)	0.695 (0.53 – 1.29)	0.60 (0.36 – 1.15)	–
HOMA-IR score	2.26 (1.51 – 3.15)	1.94 (1.28 – 2.51)	2.37 (1.59 – 6.29)	–
FIB-4 score	1.16 (0.55 – 1.5)*	0.87 (0.43 – 1.21)	1.32 (0.86 – 1.89)	0.48 (0.39 – 0.65)

ALT, alanine aminotransferase; BMI, body mass index; CRP, C-reactive protein; GGT, gamma glutamylotranspeptidase; HDL, high-density lipoprotein; LDL, low-density lipoprotein; PLT, blood platelets; RBC number of red blood cells; WBC white blood cells; WHR, waist–hip ratio.

Data are presented as median (Q1 – Q3). *p<0.05 *vs*. HCs, #p<0.05 advanced *vs*. early MASLD.

The bold values indicate statistically significant differences between the groups (p<0.05).

The study population included n=30 Caucasian patients with confirmed MASLD (16 males and 14 females) and treated at the Department of Infectious Diseases and Hepatology at Medical University of Bialystok were included in this experiment. Due to the convenience of ultrasonography, it was used as a primary means of screening the liver steatosis (17), together with serum alanine transaminase (ALT) activity (18). Moreover, a diagnosis of MASLD was confirmed with electrography, daily alcohol consumption (women <20 g/d and men <30 g/d) (19), and the absence of other causes of liver disease. No patients received pharmacological treatment at least 6 months before entering the study, in particular drugs potentially affecting cholesterol metabolism (e.g. statins, phytosterols, etc.). Exclusion criteria were as follows: cirrhotic patients, autoimmune diseases, co-infection by hepatitis B virus, or human immunodeficiency virus infections. None of the patients had systemic disorders, such as type 1 diabetes, hypertension, coronary heart disease. Patients without fibrosis and normal ALT activity were classified as early MASLD, whereas individuals with fibrosis >6.6 kPa and upper ALT activity were considered as advanced MASLD. A total of 15 healthy controls (HCs) (6 men/9 female), median age 36 (28-52), median BMI 23.2 (19.5-24.5) were included. All control subjects had no evidence or history of liver pathology.

### 2.2 Blood samples

In order to assess routinely used parameters of liver damage and function, lipid metabolism, blood glucose levels, peripheral venous blood from the ulnar vein was drawn in the morning after overnight fasting. For serum separation, samples were taken into a tube with a clotting activator and centrifuged at 3000xg for 15 min. To obtain plasma, blood samples were collected in a tube filled with EDTA K2 anticoagulant (9 mL) and centrifuged at 3000xg for 15 min. Additional plasma sample was obtained for cytokine concentration measurement and cell isolation. The resulting serum and plasma were collected and stored at -80°C (except for cell isolation) until further processing.

### 2.3 Cell isolation

Peripheral blood mononuclear cells (PBMCs) were isolated immediately after collection (ex vivo) using a Ficoll gradient as a separation system (Biochrom, Berlin, Germany). After density gradient centrifugation, the cells were resuspended in Roswell Park Memorial Institute (RPMI) 1640 medium (Sigma Chemical Co., Saint Louis, MO, USA) supplemented with 10% fetal bovine serum, 2 mmol/L L-glutamine, and 50 μg/mL. Subsequently, they were subjected to phenotypic dyes.

### 2.4 Analysis by flow cytometry

Immunophenotyping, performed on peripheral blood lymphocytes, included evaluation of IL-10 and IL-17 production by flow cytometry. To assess the frequency of regulatory T lymphocytes (Treg) in the peripheral blood, a panel of monoclonal antibodies conjugated to fluorochromes was used: anti-CD4, anti-CD25, anti-CD127, anti-IL-10 and anti-FoxP3. The developed antibody panel anti-CD4, anti-IL-17 and anti-ROR gamma (t) were used to assess the prevalence of IL-17 producing T cells (Th17 cells) in PBMC. To allow staining of the intracellular markers with anti-Foxp3, anti-ROR gamma (t), anti-IL-10, and anti-IL-17 antibodies, cells were permeabilized using Permeabilization Buffer 2 (BD Bioscience). Lymphocytes were initially identified on the basis of their morphology using relative size (forward scatter, FSC) and granularity/complexity (side scatter, SSC) properties. Following selection of the lymphocytes with CD4 marker - Th cells, further markers were analyzed for Treg and Th17 identification. The results were obtained using a FACS Calibur cytometer (BD Bioscience, San Jose, CA, USA) and analyzed with FlowJo software (Tree Star Inc., Ashland, OR, USA).

### 2.5 Anthropometric and biochemical analyses

Each patient underwent a physical examination. Measurements of weight and height were used to calculate the body mass index (BMI) [weight (kg)/height (m^2^). Waist to hip ratio (WHR) was calculated as: waist circumference was measured on the midaxillary line between the lower border of the rib cage and the upper margin of the iliac crest, and hip circumference was measured around the widest part of the buttocks. Additionally, HOMA index was determined based on formula=(glucose × insulin)/22.5 (20).

Levels of the liver function biomarkers, such as alanine transaminase (ALT), aspartate transaminase (AST), and gamma-glutamyl transferase (GGT) were assessed by enzymatic methods using COBAS INTEGRA 400 PLUS analytical platform. Blood count, prothrombin time, and prothrombin index were determined by a Sysmex XT-4000i Hematology Analyzer. Total cholesterol (TC), triglyceride (TG), high-density lipoprotein cholesterol (HDL-C) and low-density lipoprotein cholesterol (LDL-C) levels were assessed using a standard enzymatic method.

Furthermore, to assess liver fibrosis, FIB-4 index was counted based on the results: AST, ALT, platelets (in PLT morphology), and the patient’s age. We used the FIB-4 Scoring Calculator on the FIB-4 Calculator page (https://www.mdcalc.com/calc/2200/fibrosis-4-fib-4-index-liver-fibrosis). The formula: age × AST (IU/L)/platelet count (× 10^9^/L) × √ALT (IU/L) (21). In order to calculate the BARD score, 3 parameters were used: the presence of diabetes is 1 point, BMI ≥25 is 1 point, AST/ALT ratio ≥0.8 is 2 points. Scores of 0–4 were obtained, with scores of 0 or 1 having a high negative predictive value for severe liver fibrosis and scores of 2–4 was associated with a more advanced stage of liver fibrosis (21).

### 2.6 Elastography

Transient elastography with controlled attenuation parameter (CAP) is an appropriate alternative to liver biopsy and is used to diagnose and quantify the degree of steatosis with 90% sensitivity (22). Liver stiffness measurement assessed by FibroScan device (Echosens) was combined with AST, ALT, or AST: ALT ratio. Elastography was performed in the entire MASLD group (n=30). CAP cut-off values indicative of fatty liver (S) were based on (23) as follows: (1) <237 dB/m (S0, no steatosis), (2) 237.0-259.0 dB/m (S1, mild steatosis), (3) 259.0-291.0 dB/m (S2, moderate steatosis), and (4) 291.0-400.0 dB/m (S3, severe steatosis). The values for fibrosis (F) were also adopted (23): (1) <5.5 kPa (F0, no fibrosis), (2) 5.5-8.0 kPa (F1, mild fibrosis), (3) 8.0 -10.0 kPa (F2, moderate fibrosis), (4) 11.0-16.0 kPa (F3, severe fibrosis), and (5)> 16.0 kPa (F4, cirrhosis).

### 2.7 Immunoenzymatic assay (ELISA)

Plasma levels of IL-10, IL-17A, and IL-22 were measured using the Human IL-10 Quantikine HS ELISA Kit (cat. HS100C), IL-17A Human ELISA Kit (cat. D1700), Human IL-22 Quantikine ELISA Kit (cat. D2200; R&D System, Abingdon, UK), according to the manufacturer’s instructions. The results were based on the optical density of samples using microplate reader Synergy H1 (BioTek, Winooski, VT, USA). To determine the final cytokine concentration, a standard curve based on a four-parameter logistic (4-PL) curve-fit was employed.

To study the balance between Th17/Treg; Th17/resting Treg (rTreg), the ratios between IL-10/IL-17A and IL-10/IL-22 were determined.

### 2.8 Statistical analysis

Statistical processing of the collected data was performed using Statistica 13.1 (Statsoft, Tulsa, OK) and Graph Pad Prism 9 (La Jolla, CA) softwares. Statistical differences between groups were calculated, by a two-tailed Student’s t-test (if normally distributed), or if not normally distributed by Mann-Whitney U for independent variables. Data are presented as mean ± standard deviation (if normally distributed) or as median and 25%-75% percentile when appropriate. Correlations were calculated with the Spearman’s rank correlation coefficient. Differences between more than two variables were analyzed by Kruskal-Wallis ANOVA. The p values <0.05 were considered statistically significant.

## 3 Results

### 3.1 Clinical characteristics and liver function tests

A total of n=30 patients with MASLD (16 men, 14 woman) - median age 47 (38-58), median BMI [30.05 (27.77 – 33.67)] were included in this study and divided into two groups in terms of disease activity: 11 patients had early (37%) MASLD, and 19 (63%) patients had advanced MASLD, elastography proven. Mann-Whitney’s analysis of FIB-4 and HOMA-IR parameters did not differentiate between early and advanced MASLD patients. Fisher test showed statistically significant differences in BARD score between the control and MASLD groups (early: p=0.00022, advanced: p=0.00023; **Supplementary Table 1**). However, as in the case of FIB-4 score and HOMA-IR index, BARD score did not enable a distinction between the two advancement stages of MASLD (p=0.52) (**Supplementary Table 1**). Seven (23%) patients from the entire MASLD group were diagnosed with type 2 diabetes. Patients with MASLD had a higher BMI compared with HCs (p<0.001). General characteristics of the patients population and HCs is presented in **Table 1**.

In 18/30 (60%) of MASLD patients, ALT activity was elevated suggesting, together with FibroScan, the diagnosis of advanced MASLD. One of the patients had ALT in the upper limit of normal, classified in accordance with the high score elastography to the advanced MASLD group. Plasma ALT levels in the advanced MASLD group were higher [84 (58-127) U/I] compared to early stage of MASLD [38 (32-39) U/I, p<0.001]. The level of AST was enhanced in advanced MASLD and in total MASLD group compared to HCs (**Table 1**).

### 3.2 Frequencies of Treg cells and Th17 cells in MASLD patients

**Figure 1:**
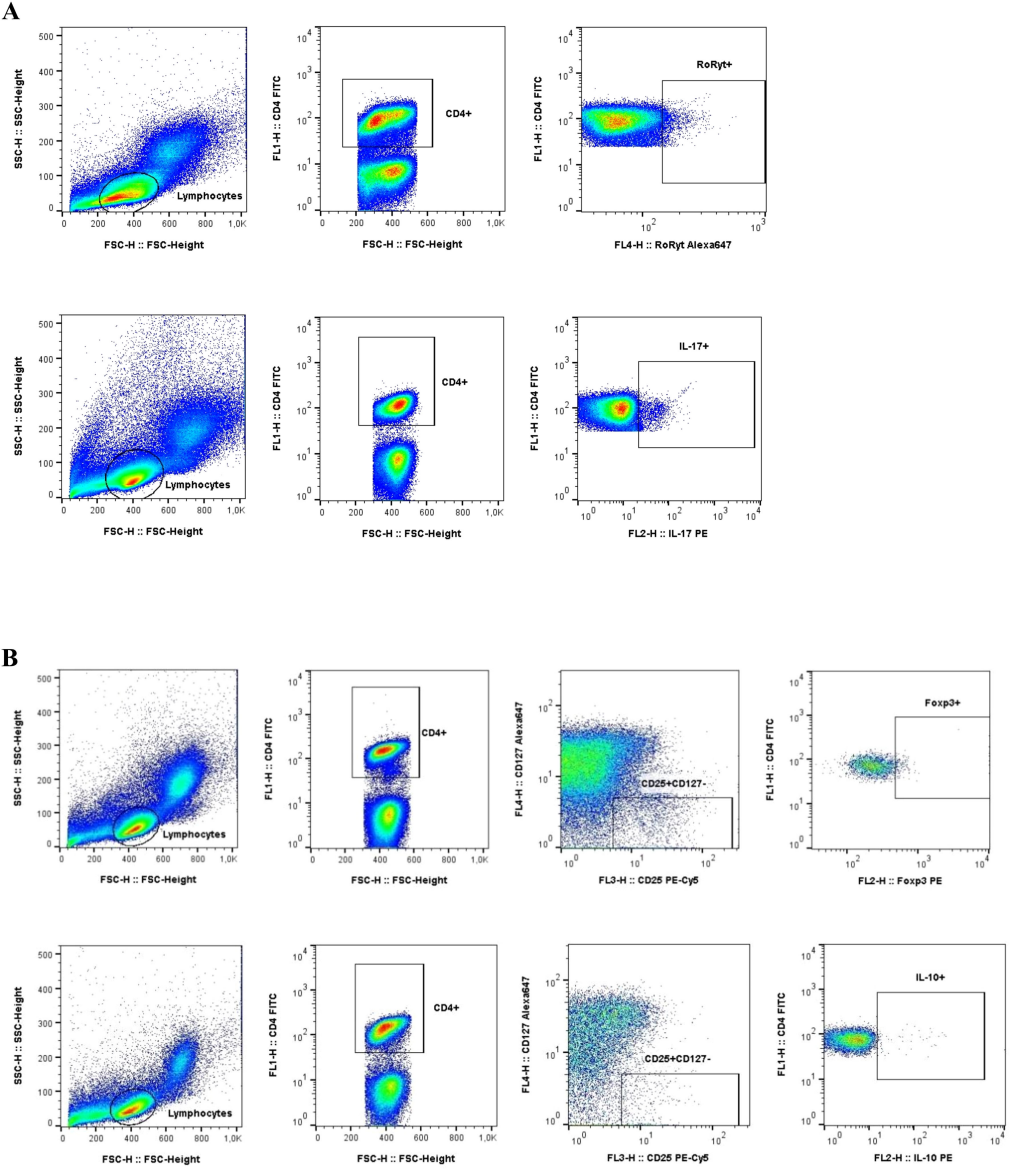
Gating strategy used in flow cytometric analysis of Th17 and Tregs cells. **(A)** Th17 phenotype was determined by the detection of IL-17 production within CD4+ lymphocytes **(B)** Tregs identification was based on CD25+CD127- phenotype and Foxp3+ expression within CD4+ lymphocytes.

The frequencies of blood Treg cells and Th17 cells in patients with MASLD were analyzed by flow cytometry. Differentiation of CD4+ T cells toward the Th17 phenotype was characterized by detection of IL-17 expression. Analysis of Treg was based on the presence of CD25+CD127- phenotype and presence of Foxp3+ expression within CD4+ lymphocytes (**Figure 1**).

The frequency of T-regulatory cells (CD4+CD25+Foxp3+, Tregs) declined in the advanced MASLD patients [3.57 (2.15-4.97) *vs* 6.56 (4.75-9.97), p=0.009] compared to the control subjects. Interestingly, we observed that Tregs Foxp3 [MFI in CD4+] and Foxp3 [MFI in CD4+ CD25+ CD127-] showed a significant increase in the early MASLD group as compared to the control group (p=0.02, p=0.005, respectively), which we did not notice in a group of advanced MASLD patients (**Table 2**). This is also confirmed by the results of inflammatory cytokine IL-10+ [% of CD4+] in the early MASLD group compared to the control group [1.73 (1.45-2.84) *vs* 1.09 (0.97-1.31), p=0.020] (**Table 2**). On the other hand, the percentage of IL-17-producing cells among CD4+ T-lymphocytes was twofold more frequent in the total MASLD group [(1.90 (0.83-3.73) *vs* 0.73 (0.54-1.56), p=0.002], which was noticed at both early [2.22 (1.59-3.66) *vs* 0.73 (0.54-1.56), p=0.007] and late stages of MASLD (1.70 (0.78-3.96) *vs* 0.73 (0.54-1.56), p=0.011; **Table 2**).

**Table 2:** Tregs/Th17 in patients with MASLD. Early and advanced stages of MASLD are compared to healthy control group (HCs).

Parameter	MASLD n=30	Early MASLD n=11	Advanced MASLD n=19	HCs n=15
Foxp3+ [% of CD4+]	3.57 (2.65-8.30)	4.67 (3.09-32.20)	3.41 (2.64-4.51)	6.80 (3.52-12.60)
Foxp3+ [% of CD4+CD25+]	4.12 (2.79-8.49)	5.16 (3.56-26.00)	3.57 (2.15-4.97)*#	6.56 (4.75-9.97)
Foxp3+ [% of CD4+CD25+CD127-]	5.03 (3.06-11.68)	7.22 (3.37-20.80)	4.62 (2.83-7.37)	9.01 (4.01-12.00)
Foxp3 [MFI in CD4+]	160.0 (50.23-223.5)	173.0 (158.0-238.0)*	75.6 (34.9-204.0)	50.1 (40.2-181.0)
Foxp3 [MFI in CD4+CD25+]	119.0 (47.9-181.3)	144.0 (98.2-209.0)	70.1 (35.4-174.0)	55.9 (39.8-153.0)
Foxp3 [MFI in CD4+CD25+CD127-]	141.0 (53.6-189.8)	163.0 (120.0-217.0)*	79.5 (41.1-175.0)	64.7 (44.7-134.0)
CD25+ [% of CD4+]	14.40 (7.22-20.13)	8.59 (5.56-14.90)	16.10 (8.92-25.90)	11.90 (7.47-21.90)
CD25+CD127- [% of CD4+]	2.96 (1.56-4.28)	3.01 (0.56-4.18)	2.90 (1.96-4.35)	2.42 (1.26-5.24)
CD25+Foxp3+ [% of CD4+]	0.57 (0.24-1.16)	0.63 (0.28-2.23)	0.57 (0.22-1.03)	1.05 (0.32-2.25)
CD25+CD127-Foxp3+ [% of CD4+]	0.15 (0.05-0.26)	0.20 (0.04-0.86)	0.15 (0.06-0.25)	0.28 (0.03-0.53)
IL-17 [% of CD4+]	1.90 (0.83-3.73)*	2.22 (1.59-3.66)*	1.70 (0.78-3.96)*	0.73 (0.54-1.56)
IL-17 [MFI in CD4+]	4.85 (3.20-6.30)	5.69 (3.64-6.58)	3.50 (3.10-5.99)	4.23 (3.41-5.38)
CD4+IL-17/CD4+FoxP3+	0.35 (0.17-0.81)	0.17 (0.11-0.51)	0.35 (0.25-1.02)*	0.11 (0.08-0.27)
IL-10 [% of CD4+]	1.43 (1.09-1.95)	1.73 (1.45-2.84) *	1.31 (1.08-1.85)	1.09 (0.97-1.31)
IL-10+ [% of CD4+CD25+]	3.08 (1.86-4.73)	3.73 (2.83-7.70)	2.49 (1.60-3.84)	3.31 (2.35-4.10)
IL-10+ [% of CD4+CD25+CD127-]	4.05 (2.88-8.01)	5.33 (3.21-8.74)	3.59 (2.26-5.28)	4.11 (2.72-5.22)
IL-10 [MFI in CD4+]	3.22 (2.80-3.99)	3.58 (3.11-4.55)	**3.00 (2.74-3.81) #**	3.23 (3.07-3.30)
IL-10 [MFI in CD4+CD25+]	3.48 (2.95-4.40)	4.17 (3.42-4.93)	3.16 (2.89-4.11)*#	3.58 (3.47-4.03)
IL-10 [MFI in CD4+CD25+CD127-]	3.44 (2.98-4.54)	3.70 (3.34-4.97)	3.28 (2.86-4.15)	3.63 (3.36-3.76)
CD25+IL-10+ [% of CD4+]	0.7 (0.4-1.2)	0.8 (0.6-1.6)	0.6 (0.3-1.0)	0.4 (0.4-0.9)
CD25+CD127-IL-10+ [% of CD4+]	0.1 (0.1-0.3)	0.2 (0.1-0.6)	0.1 (0.1-0.3)	0.1 (0.1-0.2)
IL-10 [pg/ml]	5.02 (2.97-11.90)*	3.32 (2.64-9.18)	6.29 (3.08-16.43)*	2.64 (2.24-3.34)
IL-17A [fg/ml]	235 (128-474)	123 (89-171)*	289 (208-557)*#	177 (171-300)
IL-22 [pg/ml]	**13.4 (11.6-17.8**)*	11.9 (10.5-13.4)*	16.2 (12.6-19.4)*#	8.5 (7.6-11.5)

Results are presented as median (Q1-Q3). *p<0.05 – MASLD groups *vs* control group; #p<0.05 – advanced *vs* early MASLD.

The bold values indicate statistically significant differences between the groups (p<0.05).

**Figure 2:**
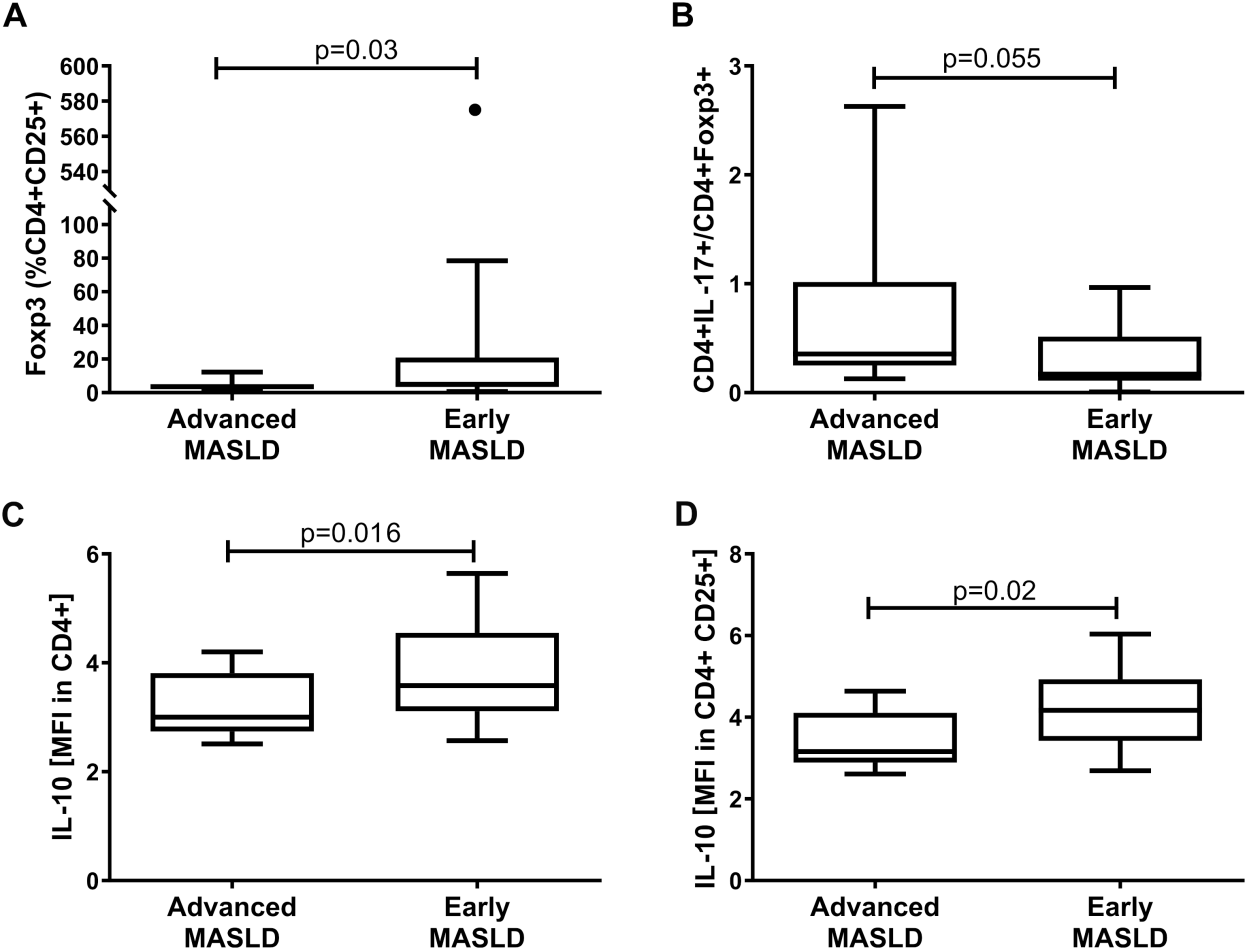
Distinguishing the group of advanced MASLD (n=19) patients from those with simple steatosis of early MASLD (n=11). Analysis of different subsets of lymphocytes: **(A)** Foxp3+ (% CD4+ CD25+), **(B)** CD4+ IL-17+/CD4+ Foxp3+, **(C)** IL-10 [MFI in CD4+], and **(D)** IL-10 [MFI in CD4+ CD25+]. Mann–Whitney U test, p<0.05.

Several parameters distinguished patients with simple steatosis at the early MASLD stage from those with steatosis with inflammation – advanced MASLD, including Foxp3+ [% of CD4+CD25+]; IL-10 [MFI in CD4+ and CD4+CD25+ populations] (**Figures 2A, C, D**). There was also a tendency toward a higher Th17 to Tregs ratio, based on CD4+IL-17+/CD4+Foxp3+ (p=0.055), differentiating advanced MASLD patients from those with early MASLD, and providing insight into the balance between pro-inflammatory and immune-suppressing responses (**Figure 2B**). Collectively, the frequencies of blood Th17 cells, based on IL-17 (% of CD4+), in MASLD patients were significantly increased compared with the HCs group (p<0.05), while Treg cell population prevalence was decreased (**Table 2**).

**Figure 3:**
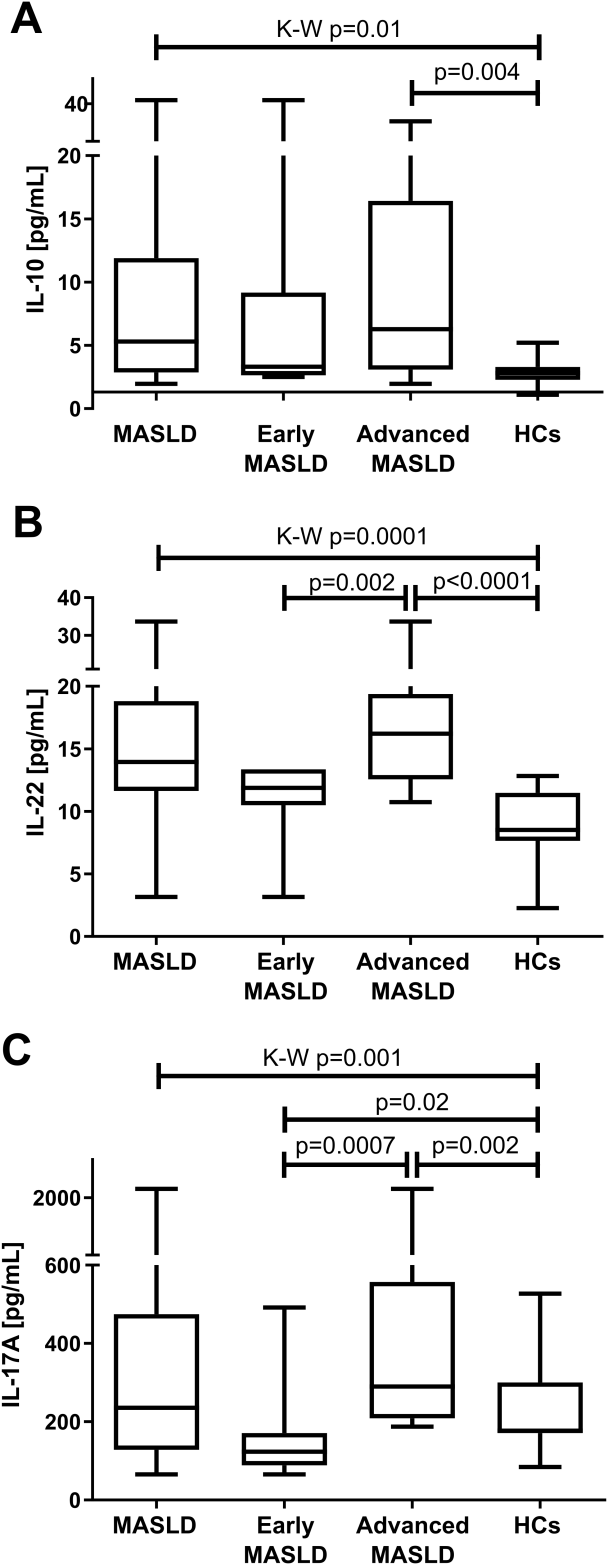
Median (Q1-Q3) plasma **(A)** IL-10 **(B)** IL-22 **(C)** IL-17A concentration in all patients with MASLD (n=30), early MASLD (n=11), advanced MASLD (n=19) patients compared to healthy controls (HCs; n=15). Mann–Whitney U test and Kruskal-Wallis ANOVA test, p<0.05.

The assessment of plasma cytokine concentration in the individual groups demonstrated significant differences in the blood level of IL-10 between patients with advanced (p=0.004) and total MASLD (p=0.003) as compared to the control group (**Figure 3A**, **Table 2**). The assessment of IL-22 not only differentiated the advanced MASLD patients from the control group (p<0.001), but also showed differences between early and advanced MASLD stages (p=0.002) (**Figure 3B**, **Table 2**). This is particularly important at the early stage of diagnosis and selection of drugs, whether the patient has a liver contusion or the inflammatory process is already underway. Interestingly, statistically significant differences were also observed in the assessment of IL-17A levels between early and advanced MASLD patients compared to the control (p=0.02 and p=0.002, respectively). Additionally, circulatory IL-17A concentration substantially raised with the progression of the disease (advanced *vs*. early MASLD, p=0.0007, **Figure 3C**, **Table 2**).

### 3.3 Correlation between Treg cells and Th17 prevalence with liver function tests and obesity-related parameters in MASLD patients

The Spearman test in the MASLD group showed that ALT activity was negatively correlated with Treg cells (IL-10 [MFI in CD4+], IL-10 [MFI in CD4+ CD25+]). Moreover, we observed a positive correlation between RoRyt+ [% of CD4+] and patients’ WHR, body weight, and BMI in advanced MASLD. Interestingly, only CD4+RoRyt+/CD4+Foxp3+ was negatively correlated with glucose in the early stages of MASLD (**Table 3**). Moreover, we showed a negative correlation between IL-10 [MFI in CD4+] and IL-10 [MFI in CD4+CD25+] with ALT in MASLD, and a positive relationship between [MFI in CD4+] and AST/ALT ratio. The strongest correlations were found between the cells from the Treg line and the parameters assessing patients’ weight. In contrast, from the measured interleukin plasma concentrations, only IL-17A correlated with the liver damage parameter (AST). We also showed a positive correlation between IL-22 and BMI in the early and total MASLD groups, as well as LDL-C and IL-22 in patients with MASLD. HOMA-IR was negatively associated with CD25+CD127-Foxp3+ [% of CD4+] and positively with CD4+RoRyt+/CD4+Foxp3+ ratio in advanced MASLD (**Table 3**). Additionally, IL-10 and IL-17A circulating concentrations exhibited negative correlations with Foxp3+ [% of CD4+CD25+], while they positively correlated with CD4+IL-17+/CD4+CD25+Foxp3+ ratio in patients with MASLD (**Table 3**).

**Table 3:** Correlations between the frequency of Th17/T-regulatory cells subpopulations, liver function tests, and obesity-related parameters (R-value by Spearman rank test).

Parameter	MASLD n=30		Early MASLD n=11		Advanced MASLD n=19	
	R	p	R	p	R	p
AST & IL-17A	0.55	**0.002**	0.21	0.79	0.25	0.36
ALT & IL-10 [MFI in CD4+]	-0.41	**0.03**	-0.26	0.44	-0.11	0.64
ALT & IL-10 [MFI in CD4+CD25+]	-0.38	**0.04**	-0.15	0.65	-0.09	0.72
AST/ALT & IL-10+ [% of CD4+]	0.37	**0.04**	0.55	0.09	0.24	0.33
AST/ALT & CD25+IL-10+ [% of CD4+]	0.36	0.05	0.71	**0.02**	0.16	0.51
AST/ALT & IL-10 [MFI in CD4+]	0.39	**0.03**	0.72	**0.01**	0.16	0.52
WHR & CD25+ [% of CD4+]	0.43	**0.04**	0.56	0.15	0.25	0.34
WHR & RoRyt+ [% of CD4+]	0.44	0.05	0.11	0.82	0.66	**0.02**
WHR & CD4+RoRyt+/CD4+Foxp3+	0.41	0.05	0.10	0.82	0.56	**0.03**
WHR & IL-10/17A	-0.48	**0.02**	-0.73	0.05	-0.26	0.33
Body weight & RoRyt [MFI in CD4+]	0.05	0.24	-0.77	**0.03**	0.43	0.09
Body weight & RoRyt+ [% of CD4+]	0.33	0.05	-0.18	0.67	0.54	**0.03**
BMI & RoRyt+ [% of CD4+]	0.32	0.13	-0.16	0.72	0.59	**0.02**
BMI & CD4+RoRyt+/CD4+Foxp3+	0.16	0.39	-0.63	**0.04**	0.59	**0.01**
BMI & IL-22	0.44	**0.02**	0.68	**0.03**	0.46	0.05
GGTP & CD4+RoRyt+/CD4+Foxp3+	0.16	0.50	0.81	**0.03**	-0.13	0.66
Glucose & CD4+RoRyt+/CD4+Foxp3+	-0.11	0.55	-0.67	**0.03**	0.16	0.50
Cholesterol & Foxp3+ [% of CD4+CD25+CD127-]	-0.52	**0.03**	-0.09	0.92	-0.12	0.73
HDL-C & CD25+Foxp3+ [% of CD4+]	-0.65	**0.001**	-0.77	**0.03**	-0.68	**0.01**
HDL-C & CD25+CD127-Foxp3+ [% of CD4+]	-0.52	**0.02**	-0.42	0.30	-0.60	**0.03**
HDL-C & CD25+ [% of CD4+]	-0.74	**0.0001**	-0.60	0.12	-0.68	**0.01**
HDL-C & CD4+IL-17+/CD4+CD25+Foxp3+	0.55	**0.01**	0.57	0.14	0.70	**0.01**
LDL-C & CD4+IL-17+/CD4+Foxp3+	0.59	**0.01**	0.29	0.50	0.69	**0.02**
LDL-C & CD4+IL-17+/CD4+CD25+Foxp3+	0.62	**0.004**	0.67	0.08	0.54	0.07
LDL-C & CD4+IL-17+/CD4+CD25+CD127+Foxp3+	0.76	**0.0003**	0.86	**0.01**	0.60	0.07
LDL-C & IL-17A	0.49	**0.03**	0.55	0.17	0.06	0.86
LDL-C & IL-22	0.74	**0.0002**	0.36	0.38	0.56	0.06
HOMA-IR & CD25+Foxp3+ [% of CD4+]	-0.38	**0.04**	-0.05	0.90	-0.56	**0.01**
HOMA-IR & CD25+CD127-Foxp3+ [% of CD4+]	-0.34	0.07	-0.05	0.88	-0.56	**0.01**
HOMA-IR & CD4+IL-17+/CD4+Foxp3+	0.48	**0.01**	0.18	0.60	0.55	**0.01**
HOMA-IR & CD4+RoRyt+/CD4+Foxp3+	0.32	0.08	-0.29	0.38	0.58	**0.01**
HOMA-IR & IL-22	0.42	**0.02**	0.14	0.69	0.41	0.08
IL-10 & Foxp3+ [% of CD4+]	-0.59	**0.001**	-0,41	0.08	-0.77	**0.008**
IL-10 & Foxp3+ [% of CD4+CD25+]	-0.60	**<0.001**	-0.51	**0.03**	-0.73	**0.01**
IL-10 & CD4+IL-17+/CD4+Foxp3+	0.55	**0.002**	0.45	0.05	0.59	0.06
IL-10 & CD4+IL-17+/CD4+CD25+Foxp3+	0.50	**0.005**	0.49	**0.03**	0.43	0.18
IL-17A & Foxp3+ [% of CD4+CD25+]	-0.42	**0.02**	-0.43	0.07	-0.02	0.97
IL-17A & CD4+IL-17+/CD4+Foxp3+	0.39	**0.04**	0.45	0.06	-0.12	0.73
IL-17A & CD4+IL-17+/CD4+CD25+Foxp3+	0.39	**0.03**	0.36	0.13	0.05	0.88
IL-22 & Foxp3+ [% of CD4+CD25+CD127-]	-0.37	**0.04**	-0.46	**0.05**	0.19	0.57

The bold values indicate statistically significant differences between the groups (p<0.05).

### 3.4 Th17, Treg cells and interleukin concentrations in the histological progression of MASLD

Advanced MASLD patients had a trend toward higher fibrosis scores compared with patients with early stages, but the difference did not reach statistical significance.

**Figure 4:**
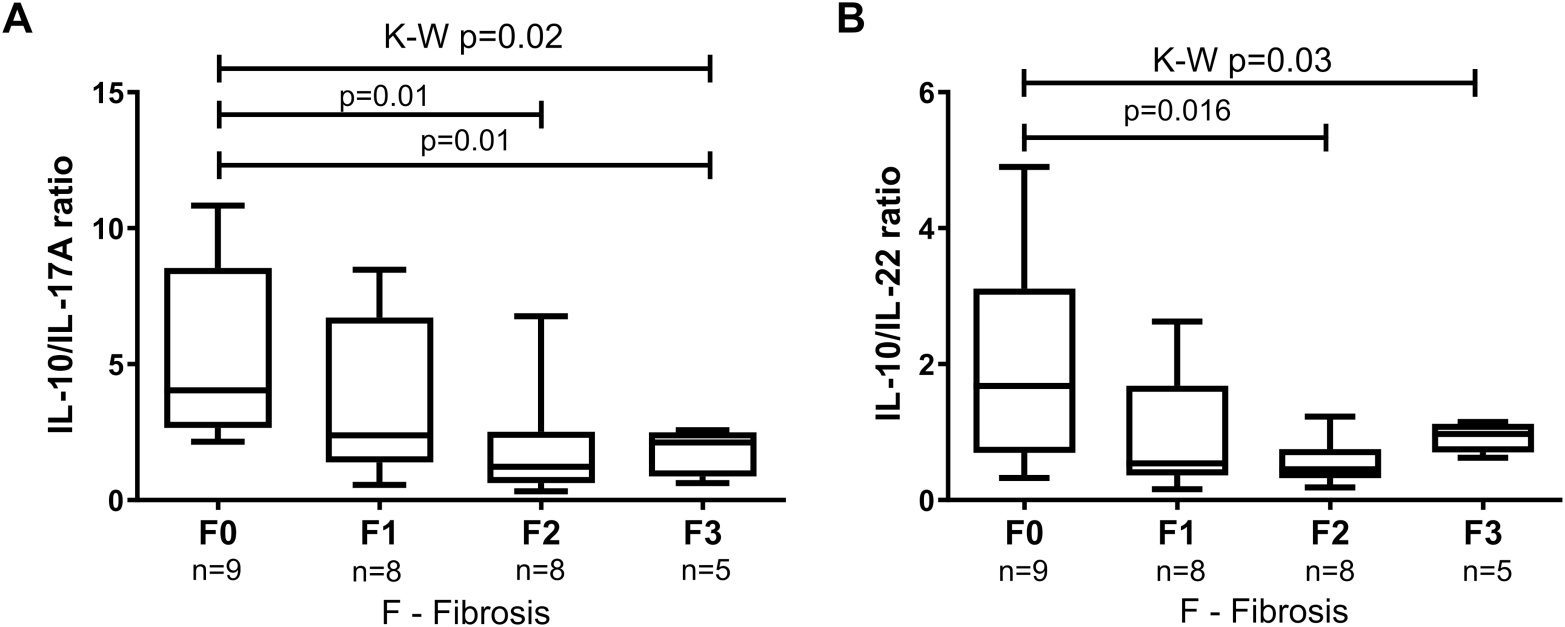
The ratio of circulatory levels of **(A)** IL-10/IL-17A and **(B)** IL-10/IL-22 in MASLD patients with subsequent grades of fibrosis in the liver FibroScan histology. Results are presented as median (Q1-Q3). Comparisons by the use of Mann–Whitney U test and Kruskal-Wallis ANOVA test, p<0.05.

Plasma IL-10/IL-17A ratio was associated with the histological advancement of liver fibrosis (Kruskal-Wallis, p=0.028) (**Figure 4**). Plasma IL-10/IL-17A ratio was higher in patients with F0 4.0 (2.7-8.5) *vs* moderate (F2) 1.2 (0.6-2.5) and severe (F3) fibrosis 2.12 (0.9-2.5) (**Figure 4A**). The plasma ratio of IL-10/IL-22 differentiates the degree of liver fibrosis (Kruskal-Wallis, p=0.03). Specifically, the highest difference was recorded between moderate (F2) fibrosis *vs* F0 (p=0.016) (**Figure 4B**).

## 4 Discussion

It is still unresolved why some patients remain clinically stable in MASLD for years while in others the disease progresses to advanced MASLD with liver fibrosis (16). Understanding the immune pathways involved is essential to determine the pathogenesis of MASLD progression, which can considerably support the diagnosis and treatment of this disease. In our study, we analyzed the composition of CD4+ T lymphocytes and assessed the concentration of interleukins from the Th17 and Treg lymphocyte lines in advanced MASLD patients compared to those with early MASLD as well as healthy volunteers. We demonstrated that the imbalance between Th17 and T-regulatory lymphocytes occurs not only at the level of serum cytokine concentration, but also in the immune response at the cellular level in the course of MASLD. Importantly, we noted the higher activity of Th17 response and IL-17 synthesis combined with lower activity of Treg cells in patients with the control group.

The prevalence of MASLD is increasing in developing as well as developed countries (24). The etiology of MASLD is multifactorial, and inflammation has been considered the main cause of the development and progression of the disease (6). Results similar to ours had been obtained earlier with mice fed a high-fat diet, where Treg/Th17 imbalance was observed causing abnormal secretion of liver cytokines that stimulate further expression of STAT3 and RORγt (6). This promotes the differentiation of CD4+ T cells, which are the main producers of IL-17A and IL-17F in the course of MASLD (24). It was demonstrated that the content of Th17 cells was elevated in mice with developed MASLD, with high IL-17 concentration in serum (6). We showed an increase in IL-17 with a statistical difference of IL-17 [% of CD4+] between the groups of advanced MASLD, early MASLD, and healthy controls. Importantly, in our study, the concentration of IL-17A in plasma distinguished patients with advanced MASLD from early MASLD group (p<0.001). Both higher frequency of intrahepatic Th17 cells, and a lower proportion of Treg cells increase inflammation of the liver, which suggests that they may contribute to the development of MASLD (6). It was demonstrated that IL-17 signaling accelerates liver cell damage and advanced MASLD progression in mice (14). Increased levels of Th17 proinflammatory cells have been reported in autoimmune diseases, rheumatoid arthritis, multiple sclerosis, chronic viral hepatitis, hepatocellular carcinoma, and Crohn’s disease (6, 25, 26). The progression from simple steatosis to steatosis with advanced MASLD inflammation is accompanied by a more visible accumulation of Th17 cells in the liver (16). In a study of 112 patients with steatotic liver disease (SLD) and MASLD, higher amounts of IFN-γ+ and IL-4+ cells were observed in CD4+ T lymphocytes in the peripheral blood of patients compared to healthy volunteers (16). Importantly, these results allowed patients with MASH to be distinguished from those with SLD by a considerably higher concentration of Th17 cells among liver CD4+ T cells (16). Our study revealed a new marker differentiating advanced from early MASLD. We demonstrated a significant difference in the level of Foxp3+ [% of CD4+CD25+], distinguishing patients with early MASLD from advanced MASLD. It should be remembered that IL-17 is induced in the course of obesity, and there is a strong correlation between IL-17 induction and inflammation and liver damage. Therefore, scientists believe that activation of IL-17 axis may lead to MASLD pathogenesis (6, 16, 24, 26–28). The aforementioned mouse model study showed that blocking the signaling pathway IL-17/IL-17RA may be a new therapeutic method to reduce hepatitis in the course of viral hepatitis (26). Reduced expression of IL-17 was observed during miR-26a administration in mice compared to the control group (29). Interestingly, in our study we observed higher values of IL-17+ [% of CD4+] in early MASLD patients than in those with advanced MASLD. The pro-inflammatory activity of Th17 lymphocytes is confirmed by the high concentration of IL-22 in the plasma of MASLD patients compared to the control group, and in patients with advanced MASLD compared to early MASLD. This may be associated with IL-17-mediated inhibition of adipogenesis by downward regulation of specific pro-adipogenic transcription factors, including CCAAT-enhancer-binding proteins (C/EBPs) and peroxisome proliferator-activated receptor (PPAR) γ (29).

Therapeutic drugs may lead to the reduction of ALT levels, but there was no positive correlation between the assessment of the histological status of hepatitis and ALT (in our case: one patient). The ongoing inflammatory process in MASLD causes an increase in ALT levels as well as IL-17 levels, which may be reduced by therapy (30). Moreover, the neutralization of IL-17 inhibited the deposition of triglycerides, and ALT level, and decreased the total mass of the liver (29). It is known that blocked signaling of IL-17 also prevents the development of hepatocellular carcinoma (HCC) (31). Therefore, it can be concluded that reducing steatosis alleviates liver damage.

Notably, Th17 cells are responsible for inhibiting the process of Treg cell differentiation (6). CD4+ CD25+ Foxp3 Treg cells contribute to the alleviation of inflammatory liver diseases (6). In the mouse-model experiment, a reduced number of liver Treg cells was demonstrated in mice with MASLD (32), and decreased expression of Foxp3 mRNA was observed in mice with advanced MASLD (15). We found significantly lower protein levels of Foxp3 [% of CD4+] and FoxP3 [% of CD4+ CD25+] in patients with early MASLD and advanced MASLD compared to higher values in the control group (p < 0.05). A similar relationship was observed in several studies with human subjects. For instance, in alcoholic hepatitis (AH), the distribution of CD4+CD25+CD127-/lo Tregs was analyzed using flow cytometry and was lower (p < 0.05) in patients with AH compared to healthy individuals (33). These results corresponded to an increased secretion of inflammatory cytokines, which is an important process in liver damage (33). Moreover, the above information implies that Foxp3 does not serve as a specific biomarker for fatty liver disease but is a more ‘pan-damage marker’.

It is probable that T lymphocytes producing IL-10 participate in the regulation of liver infection in the course of MASLD (**4**). A study that evaluated the level of Treg cells in the peripheral blood of patients with chronic hepatitis C (CHC) and MASLD/advanced MASLD, and demonstrated that the percentage of circulating CD25+CD127- and Foxp3+ was higher only in patients with CHC, and no changes were observed in patients with MASLD/advanced MASLD (34). Another study focused on the role of B-cells producing IL-10 that can control the normal development of anti-inflammatory T-cells. This suggests that not only Treg cells are significant components of immune regulation (35). Our work confirmed this hypothesis. Interestingly, the level of IL-10 [MFI in CD4+] and IL-10 [MFI in CD4+CD25+] differentiated patients with simple steatosis in early MASLD from advanced MASLD patients (p < 0.01, p < 0.02, respectively). IL-10 is a strong inflammatory suppressor as well as lowers plasma cholesterol levels in mice and humans. The use of PEG-rIL-10 in therapy decreases the level of lipids and thus may contribute to the improvement in the course of advanced MASLD (36). However, the assessment of IL-10 concentration in the blood plasma of patients did not allow for distinguishing patients with early MASLD from those with advanced MASLD inflammation; the only difference was noticed between the entire study group and the control group. Furthermore, the pro-inflammatory effect of the IL-10/IL-17A ratio was observed in patients with advanced MASLD in comparison with those without liver inflammation. IL-10/IL-17A imbalance towards an inflammatory state plays a role in the pathophysiology of steatohepatitis (13). The IL-10/IL-17A ratio observed in our study, as well as the ratio of IL-10/IL-22, varied between various degrees of hepatic fibrosis (F0-F3). The strongest correlations were observed between the lowest and moderate degrees of liver damage, with F0 degree significantly higher than F2. This may suggest a possible use of the IL-10/IL-17A and the IL-10/IL-22 ratios in the classification of patients for treatment.

It is known that different subpopulations of T cells participate in the progression of advanced MASLD. Transplantation of fecal microflora (fecal microflora transplant, FMT) aimed at changing gastrointestinal microflora is considered a new interesting treatment method. Reducing Treg cells in the liver may help transform simple liver steatosis into steatohepatitis. It was demonstrated that IFN-γ (Th1) and IL-17 (Th17) were reduced, and IL-4 (Th2), IL-22 (Th22) and Foxp3 (Treg) were increased in mice fed a high-fat diet in which the FMT method was applied. This contributed to the improvement of immune balance in the liver (37). Experiments on mice with developed MASLD showed decreased expression of Treg and Th17 cells after the intraperitoneal administration of anti-CD25 and anti-IL-17 neutralizing antibodies. The researchers found that with decreased function of Treg cells, ALT and AST levels in mice with advanced MASLD increased dramatically, with no visible histological changes in liver steatosis (15). Our results confirmed the above observations. Moreover, liver steatosis may be more susceptible to damage due to leukocyte infiltration (**4**). It should be remembered that ALT concentration increases at the moment of lipid accumulation and not after the activation of inflammatory cells (31). Therefore, it can be concluded that the progress of liver damage may be caused at the same time by steatosis, hepatitis, and insulin resistance (31).

The progression from MASLD to MASH patients can be monitored by an increased frequency of IL-17+ cells among intrahepatic CD4+ T cells, and noninvasively by a significantly higher Th17/resting Tregs (rTregs; CD4+CD45RA+CD25+) and Th2/rTreg ratio in peripheral blood (16). The above indicates heightened turnover of Tregs, potentially driven by increased activation of naïve Tregs in these patients. Therefore, our study appears useful and adequate as it was based on peripheral blood collected from patients. If the patients were stratified according to the cytokeratin 18 fragment (CK-18) level, an established marker for liver fibrosis and increased inflammatory activity, only Th17/rTreg ratio differentiated between early and advanced stages of MASLD. However, there was no significant association between CK-18 levels and Th1/rTreg or Th2/rTreg ratio in both peripheral blood and hepatic tissue (16). Future studies based on a comparison between the accuracy of the cytokine and cell-expression markers assessed in this study with other more specific immune biomarkers recently used for diagnosis and staging of MASLD (such as TIM4, TREM2, VSIG4, and FOLR2, all commonly associated with Kupffer cells) could further clarify immunomodulatory processes undergone during MASLD progression.

In conclusion, the results indicate that the pro-inflammatory state of cytokines is associated with greater histological and metabolic changes in patients with advanced MASLD. Additionally, the imbalance between Th17 and T-regulatory lymphocytes occurs not only at the level of cytokine concentration in plasma, but also at the level of cellular immune response in the course of MASLD. In patients with advanced MASLD, higher activity of Th17 response and IL-17 synthesis were observed along with lower activity of regulatory T cells. Our results emphasize the importance of Th17 lymphocyte response in MASLD pathogenesis but also suggest the potential usefulness of anti-T17 therapy in advanced MASLD. An important role is played by IL-10/IL-17A and IL-10/IL-22 relations in the progression from simple MASLD steatosis to advanced MASLD inflammatory steatosis.

## Data Availability

The original contributions presented in the study are included in the article/**Supplementary Material**. Further inquiries can be directed to the corresponding author.
