# Supplementary material for: Increased frequencies of human Th-17 CD4+ T-cells and decreased T-regulatory cells in patients with early and advanced metabolic dysfunction-associated steatotic liver disease
Source: Front Immunol. 2025 Aug 22;16:1597204. doi: 10.3389/fimmu.2025.1597204 (PMC12411424; doi:10.3389/fimmu.2025.1597204)
Supplement: Supplementary file 1 [file Table1.docx]

Supplementary Material

**Supplementary Table 1.** BARD score in early and advanced MAFLD patients, and healthy control. Fisher’s exact test.

| BARD score | Early MAFLD  n=11 | Advanced MAFLD  n=19 | HCs  n=15 |
| --- | --- | --- | --- |
| 0 | 3 | 4 | 13 |
| 1 | 4 | 9 | 0 |
| 2 | 0 | 3 | 2 |
| 3 | 3 | 2 | 0 |
| 4 | 1 | 1 | 0 |

early MAFLD vs. control group p=0.0002

advanced MAFLD vs control group p=0.0002

advanced vs early MAFLD p=0.519
